# Stability analysis of a model gene network links aging, stress resistance, and negligible senescence

**DOI:** 10.1038/srep13589

**Published:** 2015-08-28

**Authors:** Valeria Kogan, Ivan Molodtsov, Leonid I. Menshikov, Robert J. Shmookler Reis, Peter Fedichev

**Affiliations:** 1 Moscow Institute of Physics and Technology, 141700, Institutskii per. 9, Dolgoprudny, Moscow Region, Russian Federation; 2 Gero Limited, Unit 1, 12/F, International Commerce Center, 1 Austin Road West, Kowloon, Hong Kong; 3 Northern (Arctic) Federal University, 163002, Severnaya Dvina Emb. 17, Arkhangelsk, Russian Federation; 4 Central Arkansas Veterans Healthcare System, Little Rock, AR, USA; 5 Department of Geriatrics, University of Arkansas for Medical Sciences, Little Rock, AR, USA

## Abstract

Several animal species are considered to exhibit what is called negligible senescence, i.e. they do not show signs of functional decline or any increase of mortality with age. Recent studies in naked mole rat and long-lived sea urchins showed that these species do not alter their gene-expression profiles with age as much as other organisms do. This is consistent with exceptional endurance of naked mole rat tissues to various genotoxic stresses. We conjectured, therefore, that the lifelong transcriptional stability of an organism may be a key determinant of longevity. We analyzed the stability of a simple genetic-network model and found that under most common circumstances, such a gene network is inherently unstable. Over a time it undergoes an exponential accumulation of gene-regulation deviations leading to death. However, should the repair systems be sufficiently effective, the gene network can stabilize so that gene damage remains constrained along with mortality of the organism. We investigate the relationship between stress-resistance and aging and suggest that the unstable regime may provide a mathematical basis for the Gompertz “law” of aging in many species. At the same time, this model accounts for the apparently age-independent mortality observed in some exceptionally long-lived animals.

A growing number of animal species are recognized to exhibit what is called negligible senescence, i.e. they do not show measurable reductions with age in their reproductive ability or functional capacities^1^. Death rates in negligibly senescent animals do not increase with age as they do in senescent organisms. One possible example is the ocean quahog clam, which may live as much as 400 years in the wild^2^ and is the longest-living non-colonial animal. Its extreme longevity is associated with increased resistance to oxidative stress in comparison with short-lived clams^3^. No noticeable signs of aging were found in a few turtle species, such as Blanding’s turtle, whose lifespan is over 75 years^4^, and the painted turtle, which was documented to live at least 61 years. Studies indicated no differences between the mortality rates and reproductive outputs of young and old painted turtle, which is consistent with the negligible-senescence hypothesis^5^,^6^. The best-known (and arguably best-studied) example of negligible senescence is the naked mole rat, which has been documented to live in captivity for at least 28 years^7^ with no signs of increasing mortality, little or no age-related decline in physiological functions, sustained reproductive capacity over the period of observations, and resistance to common age-related diseases such as cancer, throughout their lifespans - which are at least 7 times those of mice or other rodents of comparable size^8^. These phenotypic observations accord well with exceptional resistance of naked mole rat tissues to diverse genotoxic stresses^9^,^10^. The AnAge database^11^ lists additional examples of a dozen of species that are candidates for negligible senescence.

In contrast, aging in most species studied leads to an exponential increase of mortality with age, commonly characterized by the Gompertz or Gompertz-Makeham laws^12^,^13^, which may be a direct consequence of underlying instability of key regulatory networks. Recent studies of gene expression levels in the naked mole rat and long-lived sea urchin^14^ showed that the number of their genes exhibiting expression changes with age is lower than in other animal species^2^,^3^,^4^,^14^,^15^,^16^. Therefore, the lifelong stability of the transcriptome may be a key determinant of longevity, and improving the maintenance of genome stability may be a sound strategy to defend against numerous age-related diseases. In this work we propose and analyze a simple mathematical model of a genetic network, and investigate the stability of gene expression levels in response to environmental or endogenous stresses. We show that under a very generic set of assumptions there exist two distinctly different classes of aging dynamics, separated by a sharp transition depending on the genome size, regulatory-network connectivity, and the efficiency of repair systems. If the repair rates are sufficiently high or the connectivity of the gene network is sufficiently low, then the regulatory network is very stable and mortality is time-independent in a manner similar to that observed in negligibly senescent animals. Should the repair systems display inadequate efficiency, a dynamic instability emerges, with exponential accumulation of genome-regulation errors, functional declines and a rapid aging process accompanied by an exponential increase in mortality. The onset of instability depends on the gene-network properties only, irrespective of genotoxic stress levels, and as such can be viewed as being hard-wired in the genome of the species. The two regimes also show dramatically different dynamics of stress-resistance with age: stable genetic networks are more robust against noise, and the efficacy of stress defenses does not decline with age. In contrast, the ability of “normally aging” animals to cope with stresses deteriorates with age. Moreover, the lack of stability of the gene regulatory networks may prevent complete recovery of organisms experiencing strong stresses, as can be shown by careful investigation of life histories of animals, such as fruit flies, surviving traumatic damage early in life.

## Results

### Genetic network stability analysis

**Figure 1:**
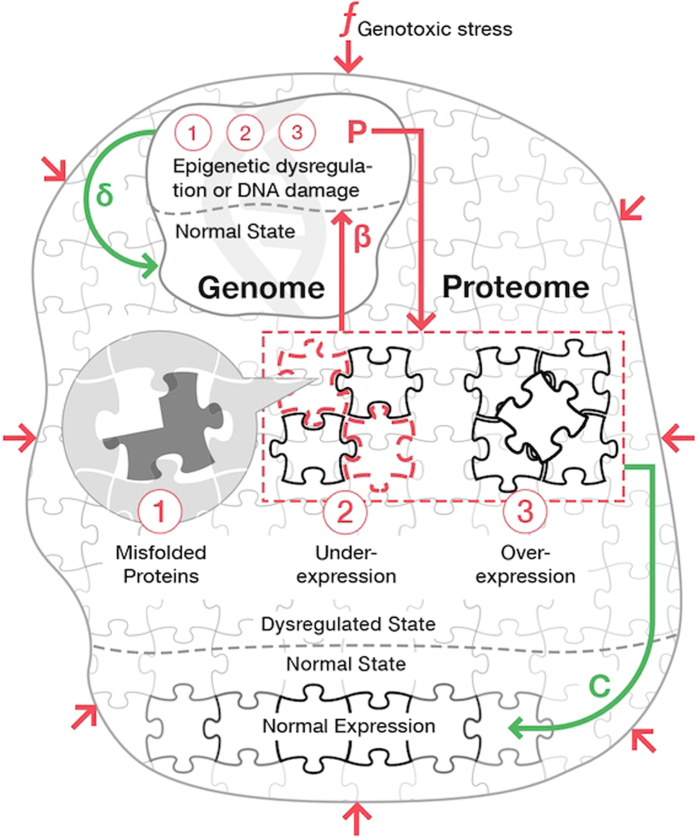
The minimum stability analysis model for a gene network. At any given time the genome consists of a number of normally expressed and dysregulated genes. The proteome accumulates “defects”, such as the proteins over- or under-expressed by dysregulated genes, which are removed via the protein quality-control or turnover systems. DNA repair machinery controls epigenetic states of the genes and restores normal expression levels. On top of this, interactions with the environment damage both the proteome and the genome subsystems, increasing the load on the protein-turnover and DNA-repair components. Parameters *f*, *β*, *δ*, *p* and *c* appear in Eqs. (1) and (2), and are interpreted in the text below. The figure was drawn by Peter Fedichev.

A living organism is an interacting system containing the genome and the expressome, defined as all the molecules (the transcriptome, proteome, metabolome) produced according to the genetic program and for which expression levels are regulated by genes and their epigenetic states in response to external influences or stresses (see Fig. 1). Likewise, the expression states of the genes are regulated by the components of the expressome. For the sake of model simplicity, but without loss of generality, we will specifically talk about the genes expressing as, and being regulated by, proteins. However, other levels of description including transcriptome, metabolome, etc. could also be viewed as relevant aspects of the expressome, similarly impacted by both endogenous and exogenous (environmental) factors. We start from the organism in a “normal” initial state, in which all genes have youthful/healthy expression profiles. With the passage of time, *t*, most of the genes retain “normal” expression profiles, while a few genes, *e*_*g*_(*t*) genes in total, subsequently become either damaged or (epigenetically) dysregulated and represent a few “defects” or “errors” in the genetic program. Gene transcripts are translated into the proteome, including its “defects”, at a certain translation rate *p*. Defects may appear in the expressome even if the genome state is perfectly regulated, due to unavoidable imperfections in translation or metabolic transformations^17^. Below, we assume that time-dependent quantities such as *e*_*g*_(*t*) may be averaged over times longer than the characteristic interaction times, but are still much shorter than the lifespan of the organism.

Next, we assume that the initial state of the genome is almost stable, meaning that the number of improperly expressed genes is small relative to the total genome size, 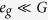 and, therefore, the number of improperly produced proteins copies, *e*_*p*_(*t*), is also relatively small. This allows us to ignore the interaction between defects in the genome and the proteome. Accordingly, the most general model describing the dynamics of the interacting defects in the genome and in the proteome can be written in the following form:


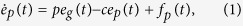



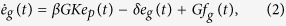


Here 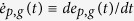, *β* is the coupling rate constant characterizing the regulation of gene expression by the proteins. The constant *K* is the average number of genes regulated by any single protein and represents a simple measure of the overall connectivity of the genetic network. The constant *c* reflects the combined efficiency of proteolysis and heat shock response systems, mediating degradation and refolding of misfolded proteins, respectively, whereas *δ* characterizes the DNA repair rate. The parameters—coefficients in Eqs. (1) and (2) are described by arrows on Fig. 1. Furthermore, the model includes the “force” terms, *f*_*p*_(*t*) and *f*_*g*_(*t*), which characterize the proteome and genome damage rates, respectively. The forces can represent any of a number of things, including oxidative stress (metabolic), temperature, gamma-radiation (environmental), that are imperfectly compensated by protective mechanisms.

Eqs. (1) and (2) can only hold in their simple linearized form if the total number of regulatory errors is small and the defects do not interfere with the repair machinery or any other rare essential subsystems of the gene network. If, for example, a defect alters a DNA repair system-associated gene expression or protein level, the repair rate drops, the system becomes unstable and may quickly diverge from its normal state^18^,^19^, as we show below. To avoid complications arising from introducing such nonlinearities, we adopt a simple hypothesis as to how defects in the evolving gene network could be responsible for the demise of a cell or organism. More specifically, we assume that mortality at any time is dependent on the probability of a defect to “land on” and damage or dysregulate an essential gene in a sufficient fraction of cells to lethally impair tissue function locally. Any gene in the model can be dysregulated for a short time (brief relative to lifespan) and then “repaired”, or its products acquired from healthy neighboring cells. Therefore, a gene is considered essential if the disruption of its expression, even for a limited time, is lethal to cells in which it was disrupted, once their occurrence exceeds some threshold fraction of cells. The suggested picture is quite general, however, and is easily extended to entire animals, since the stability boundaries are the same—only the exponents will be reduced because some threshold fraction of a tissue must die (in some most-vulnerable tissue type) to produce organism lethality. In this case the population dynamics of a set of gene networks representing *N*(*t*) organisms can be represented by:


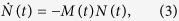


where *M*(*t*) = *ωe*_*g*_/*G* is the mortality rate, proportional to the fraction of mis-regulated genes, *e*_*g*_/*G*, and *ω* is an empirical factor, roughly a measure of the (small) fraction of genes in the whole genome that are essential.

As shown in Methods, a general solution to Eqs. (1, 2) is a linear combination of two functions characterized by well-separated time scales. Most external perturbations lead to responses, which relax quickly at time scales on the order of the network’ s inverse relaxation rates, *c* and *δ*. However, life-long changes, such as aging or development, are usually considerably slower. Therefore we can use adiabatic approximation to obtain the effective equation for the age-dependent changes in the number or regulatory errors and, consequently, the mortality


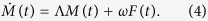


Here *F* = *f*/(*c* + *δ*) is a combined measure of genotoxic stress, 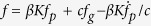, and Λ = (*βpGK* − *cδ*)/(*c* + *δ*) is the exponent characterizing genetic-network stability, which is precisely the propagation rate of gene-expression-level perturbations. As we will see later, in the long run, stress levels can be averaged over longer periods and hence presumed to be time-independent, *f*_*p,g*_(*t*) = *const*. This yields the following expression for the age-dependent mortality rate:


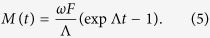


The nature of the solution is very different depending on the sign of the exponent Λ. Whenever the combined efficiency of all repair systems is lower than a measure of the defect proliferation rate,


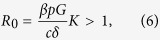


then the gene network is unstable, Λ > 0, and the number of regulatory errors (defects) in the genome and in the expressome grows exponentially along with mortality, *M*(*t*) ∼ exp(Λ*t*), which is precisely the celebrated Gompertz law^12^. The Gompertz exponent, Λ, is related to the Mortality Rate Doubling Time (MRDT), *t*_Λ_ = ln 2/Λ, whereas the average lifespan is given by


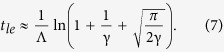


The quantity depends on both the exponent Λ and on the genotoxic stress level through the parameter *γ* = *M*_0_/Λ, where *M*_0_ ∼ *ωF*/Λ is the mortality at birth or Initial Mortality Rate (IMR) in our model. For many species following the Gompertz mortality law *γ* is very small (*γ* ≈ 0.05 for fruit flies if computed using MRDT and IMR values from AnAge database^11^). Accordingly, the logarithm is usually large and the life expectancy, *t*_*le*_ ≈ Λ^−1^ log(1/*γ*), greatly exceeds MRDT, the time scale characterizing the gene network instability. This is our precise definition of the Gompertz limit describing long lived species including humans, when the logarithm of a large argument is a very slow function, and the lifespan of the species is determined by the gene regulatory network properties only and depends very weakly on the genotoxic stress level through the value of *γ*. This also means that the lifespan does not depend on precise specification of stress levels or their variation over time. The same argument would hold if a small non-linearity is added in Eq.(4) and thus establishes a considerably wider applicability range for the basic linearized Eqs. (1, 2, 3).

A considerably more intriguing situation may occur when the genome is stable, Λ < 0 or *R*_0_ < 1, and the gene network may remain stable under reasonable stress conditions for a very long time. The fractions of dysregulated genes and of misexpressed components of the expressome will then stabilize at constant levels, as will the mortality rate itself maintain the same level 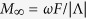. Constant mortality rate means that the population of animals dies off exponentially rather than age-dependently: 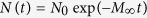, which is much slower than the Gompertz-law prediction. We believe that the age-independent mortality observed in naked mole rat experiments over a very long lifespan^7^, together with exceptional stress resistance of naked mole rat tissues^9^, may be manifestations of this stable scenario. We predict that the gene networks of negligibly senescent animals are exceptionally robust, and the number of dysregulated genes will scarcely change with age. This argument is supported by the observations^16^ in which the number of genes differentially expressed with age was compared among naked mole rat, mice and humans.

### Aging of the fruit-fly transcriptome

**Figure 2:**
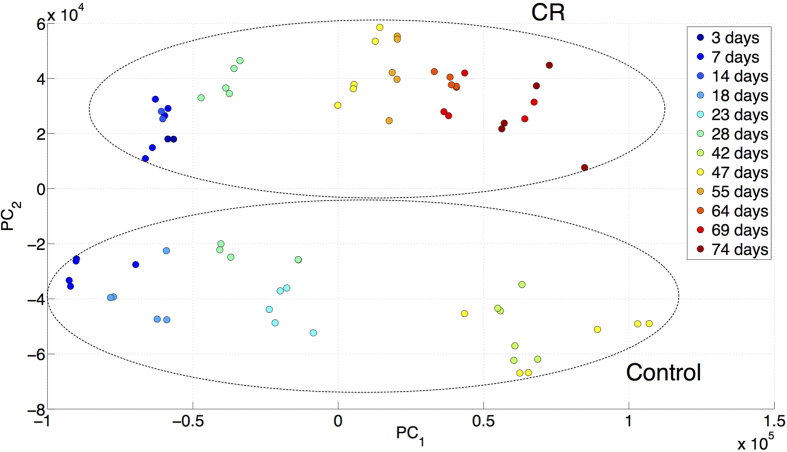
Principal components analysis of gene expression profiles in aging flies (data from^20^), fed on control (ad lib) and Calorically Restricted (CR) diets. Every point represents a transcriptome for flies of a specific age and diet. As the animals age, the genetic network accumulates regulation errors and the transcription levels change in a single direction, up to a limit beyond which viability cannot be maintained.

Analysis of model solutions (see Methods) suggests that aging in gene networks of “normally” aging or Gompertzian animals manifests itself as a highly correlated changes to the genome and the expressome states, occurring on distinct and well-separated time scales. We show that even though most external stresses lead to perturbation of the gene network, which relax quickly back to unperturbed levels, many experimentally measurable properties of the organism state should reflect the underlying instability, to an increasing degree as animals age. This means that gene-expression (or metabolite, etc.) levels should change with age in a coordinated manner and slowly deviate from their healthy/youthful states. We then asked whether our model is supported by gene-expression data from fruit flies (ref. 20). The measurements were performed at 6 different ages, for two groups of adult *Drosophila melanogaster*: normally (“ad lib”) fed control flies and calorically restricted (CR) flies. Figure 2 is a Principal Components (PC) analysis plot, in which each point represents the state of gene expression for one combination of age and diet.

Remarkably, aging in flies follows a unidirectional and thus apparently pre-defined or hard-wired in the genome trajectory of gene expression (along PC1) throughout their lifespans, accompanied by apparently rapid and random expression changes along orthogonal directions such as PC2. Variance along the PC2 axis is nevertheless small relative to the inter-group differences distinguishing ad lib from CR-fed flies. This may indicate that the corresponding transcriptional changes occur in response to, for example, nutrient-supply variation. Along the PC1 dimension, although there are stochastic contributions, there is a strong, systematic dependence on age in each of the two diet groups. Points on the extreme left correspond to the youngest flies, and points for older age-groups are displaced progressively to the right. Thus, deviation of the gene expression profile from the young state increases with age, indicating that the number and extent of dysregulated genes increases along with mortality up to a point when the accumulation of gene-expression abnormalities becomes incompatible with survival of the organism. This interpretation goes well in hand with the arguments used in derivation of Eqs. (1, 2, 3) and qualitatively support the presented model as a very general description of aging in gene networks of realistic animals, including multicellular organisms, such as fruit flies. We describe a generalization of the equations as well as the transcriptional and metabolic changes in aging flies along with their relation to the Gompertz mortality law in a subsequent work^21^. Both of the groups age in a similar way (along the same PC1 direction), but at a considerably different rate. We are leaving the detailed analysis of the aging trajectories differences between CR-fed and control flies for a future work.

### Genetic-network stability and stress resistance

**Figure 3:**
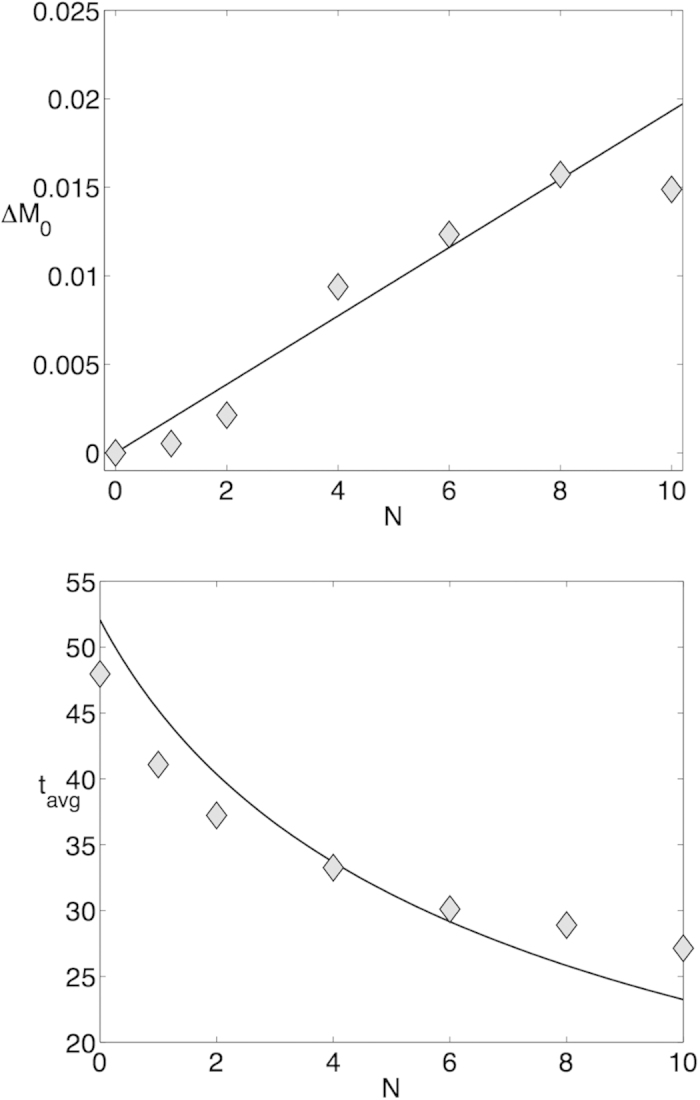
(**a**) Δ*M*_0_, the mortality change as wild-type flies are exposed to *N* traumatic insults, is plotted as a function of *N*. (**b**) Average lifespan, *t*_*avg*_, is plotted as a function of *N*. In both panels (**a**,**b**), the solid lines indicate the theoretical prediction based on Eq. (6) and parameters estimated from experimental data. Grey symbols are experimental mortality data points.

The proposed model may be considered as a general theory that subsumes previous “error catastrophe” theories^19^,^18^ as special cases. It was long considered that error catastrophes can be probed in experiment where the effects of various stresses on animal lifespan were observed^22^. To understand how the model presented above deals with stresses, we will first reanalyze a related experiment from^23^, in which flies of varying age were exposed to a traumatic brain injury (TBI) for a short time and then observed for a time *T* that is small compared to the lifespan of the animals. The mortality index *MI*_*T*_ is calculated as the fraction of animals alive at the start of stress, dying over a short observation interval during or following stress application. The post-stress lifespan was also determined. To model the experimental settings we assume that the animals at an early age *t*_0_, 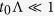, were subjected to an external genotoxic stress characterized by the amplitude *F*^*s*^, which is proportional to number of traumatic strikes *N* in each experiment. As shown in Methods, a generic stress perturbs the state of the gene network and within a linear response theory, the mortality in the experiment has a contribution to both the slow and fast modes. At late ages, the influence of the stress mostly dies out due to the fast relaxation processes in the genome and the proteome. On the contrary, since the gene network of flies is unstable, the influence of a stress applied early in life is maintained in the slow mode and shortens the lifespan of the animals. The mortality *M* as a function of time *t* and of number of traumatic events, such as strikes, *N* is given by


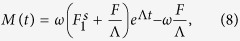


where 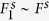 is an empirical factor, proportional to the number of the strikes, 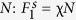, where χ is a stress and a species-dependent constant. Accordingly, we predict that the difference Δ*M*_0_ between mortality in flies, exposed to a different number *N* of traumatic strikes, and mortality of the flies in the control group is proportional to *N* at any given age. Figure 3a shows a direct comparison of the mortalities of the treated and the control groups obtained from our analysis of the data from^23^. An alternative way to see this is to calculate the model prediction for the average lifespan as a function of *N*


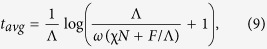


and compare it with the experimental lifespan in groups with various stress levels. The results of the analysis of the population dynamics data from^23^ are presented in Fig. 3a,b, and show a fair agreement between the dependences observed in the experiment and the simple model predictions.

Even though the mortality at late ages does not depend on the fast gene network dynamics, the Mortality Index itself contains both kinds of contributions,


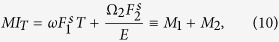


where *E* = (*c* + *δ*) is the effective relaxation time in the expressome (see the analysis leading up to Eqs. (15) and (19) in Methods). Empirically, 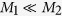 in TBI experiment and therefore very strong stressors are required to produce a measurable change in the lifespan. This may be extrapolated to and compared with findings reported earlier^22^, where Drosophila adults in experimental groups were treated for 3–5 days with a number of agents shown to increase misincorporation into protein or RNA, at doses leading to <20% mortality. Although these treatments produced error rates much higher than were seen in the course of aging in control flies, and produced a small mortality increase during the treatment, the misincorporation rates subsequently returned to control (pre-treatment) levels. This would be consistent with predictions of our model, if most of the stress-induced perturbations relaxed quickly to a level nearly indistinguishable from controls. Because the overall Mortality Index in the experiment was small, the average lifespans of survivors were indistinguishable from controls^22^. The effects of the stress on the lifespan may have been further reduced by hormesis, a known ability of weak stresses to improve survival of animals^24^,^25^,^26^. The latter is clearly a non-linear phenomenon, which is not very strong in the Gompertz limit in any case and can not be explained by the suggested simple linearized model.

Extreme longevity has long been associated with exceptional resistance to a variety of stresses^27^. And conversely, the decrease of stress resistance with age is one of the best-established indices of aging. The relation should be taken with caution, since the stress resistance measured by the Mortality Index and described by Eq. (10) contains the contribution of processes occurring at all time scales. Only a measure of stress resistance associated with the response of the slowest modes of the gene network can be related with aging and longevity. A curious situation may occur when Λ < 0 in Eq. (6), indicating that the efficacy of repair systems is high enough to prevent exponential system deterioration with age. This would also imply robust resistance to stresses, consistent with the exceptional stress resistance of negligibly senescent species^9^. For example, a comparison of survival between negligibly senescent vs. short-lived clam tissues treated with tetr-butyl hydroperoxide showed much higher resistance to oxidative stress in long-lived clams^3^. Also Oxygen Radical Absorbance Capacity (ORAC) was measured in young and old clams of both types. The experiment showed an age-related decline in ORAC for shorter-lived clams, whereas ORAC did not change with age in tissues of negligibly-senescent clams, which is entirely in line with the model predictions, since there are no possible changes in the gene network state leading to a deterioration of the genotoxic compensation abilities, if the gene network of an organism operates in the stable zone.

## Discussion

Eq. (6) implies many possibilities to stabilize a regulatory network and thus extend lifespan, as summarized in Table 1. According to Eq. (7), a reduction in genotoxic stress levels by isolation of genes from the environment can protect from direct stresses but can only produce a weak (logarithmic) increase in lifespan. Much stronger effects could be achieved by interventions aimed at increasing gene-network stability and hence reducing Λ. This can be accomplished, for example, if genes and their epigenetic states were isolated from regulatory signals, which equates to decrease in *β*. This could be the reason why extraordinary longevity in C. elegans is accompanied by silencing of most signaling pathways in particular pathways comprising kinase cascades that most often lead to activation or inactivation of transcription factors^27^,^28^. Such defensive strategies appear to have been utilized during the course of evolution, e.g., in protecting mitochondrial genes by their transfer to the nuclear genome, and by establishment of the nuclear envelope, considered a major factor leading to the emergence of multicellular life.

Turnover of the proteome or metabolome, *c*, and repair efficiency, *δ*, are factors shown to modulate lifespan in several species. Indeed, DNA repair pathways are encoded by hundreds of genes^29^ that are variously involved in detection of DNA damage, enzymatic manipulation of damaged DNA, and homologous recombination between DNA strands, often permitting complete restoration of the original sequence even when portions have been lost from one chromatid or chromosome homolog^30^. A somewhat more surprising result is that both the proteome and genome repair rates, *c* and *δ* respectively, formally contribute to the result on an equal footing. This means that increasing protein turnover may help protect against DNA damage and *vice versa*. Increased protein turnover rates, as can result from increased ubiquitin-proteasome activity, have been demonstrated to result in increased longevity in yeast. Enhanced proteasomal activity confers a 70% increase in median and maximum replicative lifespan, comparable to the effect size for many single-gene mutations identified, and greater than the extension observed by deletion of TOR1 or over-expression of SIR2^31^.

Recent data reveal that the naked mole rat has highly efficient protein degrading machinery and thereby maintains high levels of protein quality control, constantly degrading misfolded and damaged proteins, thus maintaining uniform steady-state levels throughout life^32^. Our model predicts that DNA repair efficiency, *δ*, is as important for lifespan extension as increases in the protein turnover rate, *c*. These naked mole rat results are interesting because their exceptional longevity occurs despite the presence of chronic oxidative stress even at young ages^9^,^33^. The relation between protein homeostasis maintenance efficacy and aging highlighted here in view of the model findings has been previously brought up in a number of works using very different approaches, see e.g.^34^,^35^,^36^.

Cell division is a trivial way to rebuild the cell components and dilute expressome defects in half (symmetric division) or more (asymmetric division), especially relevant to yeast and continuously dividing tissues. This simple dilution principle links the protein turnover rate to cell division frequency, so that decreasing the protein turnover rate *c*, by inhibiting cell-cycling or by other means, may be used as a gene-network destabilization strategy for anti-cancer and antibiotic treatments^37^.

In differentiated organisms there appear even more ways to maintain stability of the gene network. Metazoan (multi-tissued) animals have the ability to eliminate cells that have sufficiently damaged or unstable genomes, in a process called apoptosis, and replace them with healthy, stem cell-derived cells. Thus the repair rates *c* and *δ* also cover the contributions of these apoptotic and regeneration pathways. Moreover, as cells divide and differentiate to form new tissues and cell types, resulting in many different epigenetically stable states of the same genome, all of the model constants can also vary depending on the tissue involved. Thus our model clearly permits the instability and aging rates of different tissues to vary. This does not, however, alter the outcomes, which will reflect the vulnerability of the most unstable tissue on which animal survival depends - most probably, the stem-cell subsystem corresponding to the most renewal-dependent tissue within the body.

According to the stability requirement (6), large genomes are difficult to maintain. There are multiple ways to increase system stability by limiting the size of the expressed genome. One such strategy, clearly of ancient origin, is differentiation, wherein only a small fraction of the full genome is expressed by any one cell type at any point in time. Another possible way to regulate the stability of the genetic network is by modulating the degree of network connectivity. The robustness of a simulated gene network with respect to external noise was recently shown to be associated with the connectivity of the network^38^. This compares very well with our stability condition Eq.(6), which predicts that a gene network becomes stable if the characteristic measure of network connectivity becomes sufficiently small: *K* < *K*_0_ = *cδ*/(*pβG*). A recent study of long-lived *Myotis brandtii* genomes found a wide range of genetic abnormalities in the GH/IGF1 axis^39^. Ablation of growth hormone signaling may produce a reduction of the effective network connectivity *K*, which thus could be one of several possible explanations for the inordinate (relative to size) longevity of bats. All the above strategies are mathematically equivalent and exist in nature; indeed, there are cases in which multiple strategies are employed to increase lifespan^39^.

The presented work concerns a simplified version of an aging model, which includes the genome interacting and being regulated by different expressome components, such as transcriptome, proteome, metabolome etc. In a subsequent work^21^ we show that the errors dynamics and the characteristic Gompertz mortality can be carefully derived as a mean field approximation to a more general model describing critical dynamics of realistic gene networks. In fact the generalization corroborates our fundings and proves that the simple theory provides a correct semi-quantitative description of aging in the Gompertz limit. The extended model lets us establish signatures of aging and all causes mortality in transcriptomes and metabolomes. We leave further analysis of aging in multi-omics datasets, possibly including measurements from different tissues of a single organism and generalization to other species such as humans for a future work. We believe that subsequent developments of the presented theory of aging should once day help to produce useful markers of aging and probably genes, regulating aging in realistic organisms.

## Conclusions

In summary, we have provided a mathematical explanation for the dramatic variance in lifespans seen in the animal kingdom, relating this variance to genetic-network stability and resistance to stresses. We developed a model, which lets us define Gompertzian or “normal” aging as an exponential accumulation of gene-regulation abnormalities rooted in the inherent instability of gene networks occurring under most common circumstances, and causing a progressive loss of stress resistance along with the characteristic mortality increase with age. This, in turn, produces susceptibility to age-related diseases and hence can be viewed as causing their onset. We show that in a very well defined Gompertz limit only, when the mean lifespan of the species exceeds the mortality rate doubling time by a sufficiently large margin, the gene regulatory network instability occurs depending on the genetically encoded factors only irrespective of the environmental stresses, which is consistent with the programme of aging hypothesis^40^,^41^. Ultimately, ever increasing number of regulatory errors is responsible for the death of an organism. This seems consistent with previously reported experimental mouse data indicating that epigenetic dysregulation contributes far more (by up to two orders of magnitude) to the loss of gene-expression integrity than somatic mutations alone^42^. On the other hand, genetic networks of animals with better transcription fidelity or other mechanisms of genome-maintenance are not only more stress-resistant, but under specific conditions may become stable and produce the phenotypes we call negligible senescence^43^. An even more important corollary is that the gene networks of extremely stress-resistant animals (extremophiles) can still be unstable, because network stability is determined by the value of the combined parameter *R*_0_ given by Eq. (6), and not directly by high *c* and *δ* rates.

The most important results of this study are Eq. (4), phenomenologically describing aging of a gene network, and the concept of fundamental genomic instability described by Eq. (6). We show that the lifespan of a species is determined by the stability of its most vulnerable gene network. Mathematically, there exist two types of solutions to Eq. (4), implying the possibility of both stable and unstable gene regulatory networks. We posit that the stable genomes correspond to negligibly senescencent animals, which hence are robust with respect to environmental or endogenous noise. Although unstable gene networks exponentially accumulate gene expression errors and eventually disintegrate, the growth exponent is nevertheless small and the characteristic mean lifespans can be made sufficiently large and weakly dependent on genotoxic stress factors in the Gompertz limit. This may be an explanation of the apparent prevalence of Gompertzian aging in Nature. We are able to show that the lack of stability of realistic gene regulatory networks may manifest itself in experiments in the form of incomplete recovery of organisms experiencing strong stresses, which can be established by careful investigation of life histories of animals, such as fruit flies, surviving traumatic damage early in life. Since gene networks stability is naturally related to aging and can be favored in multiple ways, further research has the clear potential to create novel therapies to protect against the most morbid age-associated diseases, and perhaps even against aging itself.

## Methods

### Derivation of Eq (4)

**Figure 4:**
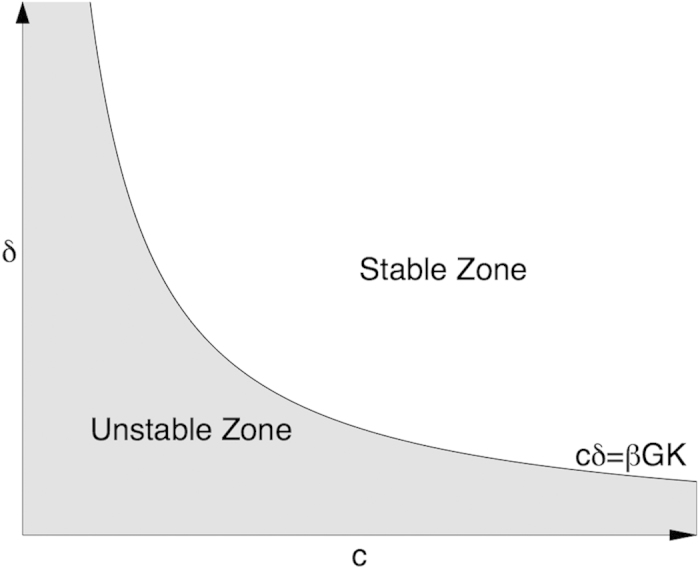
Stability diagram of the model gene network. Below the separatrix defined by Eq. (6) the solutions of Eqs. (1,2) are unstable and correspond to “normally” aging animals. The stable solutions exist above the separatrix and may describe “negligible senescence”.

Eqs. (1) and (2) can be formally represented in the matrix form


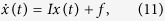


where *x*(*t*) = (*e*_*p*_, *e*_*g*_)^*T*^ is the full state vector, characterizing both the genome and the expressome variables in the model. The matrix


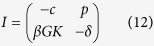


describes the interactions within and between the genome and the interactome components, while the “force” term, *f* = (*f*_*p*_, *Gf*_*g*_)^*T*^ represents the combined action of genotoxic factors, which, for simplicity only and without loss of generality, we presume to be time independent, *f*_*p*_, _*g*_(*t*) = *const*. The general solution of the linear system can be written as


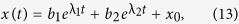


where λ_1,2_ are the two eigenvalues, and *b*_1,2_ are the corresponding right eigenvectors of the interactome matrix *I*. The vector *x*_0_ is the “integration constant” to be selected to match the initial conditions. Since the state vector in the model contains the number of regulatory errors, which is presumed to be initially small, i.e. at young ages, we choose *x*_0_(0) = 0. The eigenvalues of matrix *I* are


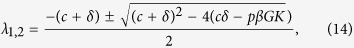


and are always real. Both values are negative and hence correspond to stable solutions, provided the genome and the proteome-maintenance system efficiencies are sufficiently high. This corresponds to area above the phase boundary, the separatrix line *βpGK*/*cδ* = 1, on the stability diagram (see Fig. 4).

Below the separatrix line on the stability diagram, one of the eigenvalues becomes positive and, in the long run, dominates the solution (13). The gene network is unstable in this case and therefore the separatrix defined by Eq. (6) is the bifurcation line separating two domains. Close to the separatrix the instability rate is small and hence the lifetime of the gene network is large. Accordingly, the eigenvalues can be represented as Taylor series


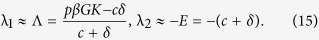


Since the number of the defects in the gene network in our model is directly related to mortality by Eq. (3), the smallest eigenvalue, *λ*_1_, is exactly the instability rate in Eq. (5) and is proportional to the inverse mortality rate doubling time (MRDT). Therefore the slow dynamics of the gene network are associated with aging and are dominated by a single mode, a correlated change of the genome and the expressome along the “direction” *b*_1_. The other eigenvalue, *λ*_2_ ≈ −*E* = −(*c* + *δ*), is still negative. The characteristic time scales responsible for repair and relaxation in the genome and the proteome are normally shorter than the lifespan of an organism, and therefore the eigenvalue is large, 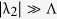. Accordingly, the corresponding part of the solution (13), the coordinated dynamics of the network state along the vector *b*_2_, describes fast relaxation processes in the gene network.

In fact, the presented derivation can be made a part of a more general argument. Eq. (1) can be formally solved as


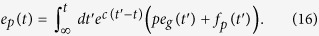


Close to the stability-instability boundary, the gene network dynamics have a slow mode and therefore the age-dependent changes in the system variables are slow compared to the characteristic relaxation time in the expressome. Accordingly, the integral quickly converges and the number of regulatory errors in the expressome can be formally expanded as: 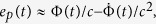 where Φ(*t*) = *pe*_*g*_(*t*) + *f*_*p*_(*t*). The expansion works if the age-dependent changes are slow compared with the proteome turnover rate, 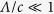, a condition that definitely holds for most species. Substituting the expansion for *e*_*p*_ into Eq. (2) we obtain Eq. (4). Remarkably, the derivation would even work if the expressome vector has any number of components, i.e. describes states of multiple proteins or metabolites, provided, of course, that the relaxation times associated with all components are small compared to Λ^−1^. Therefore Eq. (4) can be used to describe the slow dynamics of the gene network close to the bifurcation point in more complicated models (an in-depth analysis that will be presented in a subsequent publication).

### Derivation of Mortality Index

Assume that a certain toxic stress or agent is acting on a group of animals for a short time at a young but not immature age (i.e., at an age smaller than the inverse mortality doubling time ∼Λ^−1^). In this case, the experiment can be described by the generalization of Eq. (11)


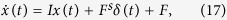


where the new vector quantity *F*^*s*^ characterizes the toxic stress. Physically the components of *F*^*s*^ are proportional to the number of errors produced in the network by the stress factors and hence are proportional to the duration and intensity of the stress. The latter can be characterized by the number of mutations or strand breaks produced by radiation, or number of proteins misfolded due to a temperature change, depending on the experimental setup. Close to the bifurcation point, the solutions of the equations are


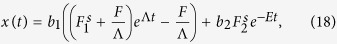


where 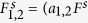), and *a*_1,2_ are the left eigenvectors of *I* (we employ the normalization condition (*a*_*i*_*b*_*k*_) = *δ*_*ik*_). Assuming that mortality is produced both by the errors in the genome and in the expressome, in the linear approximation we have *M*(*t*) = (Ω*x*), where Ω = (Ω_*p*_, Ω_*g*_)^*T*^ are organism specific quantities. If the stress factors are applied for a short time, the Mortality Index, or the fraction of animals dying within the observation time interval *T* following the stress, 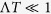, can be expressed as


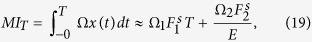


where Ω_1,2_ = (*b*_1,2_Ω). Experimentally, *MI* is dominated by the fast-mode contributions, which means that projections of realistic stressors on the slow mode components small, 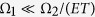.

At late ages, 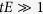, the fast mode in Eq. (18) dies out and the mortality of the survived animals follows a variation of the Gompertz law


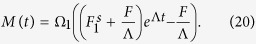


Comparing the expression with Eq. (5) describing the mortality in the control group we find that 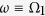. Since the slow dynamics are unstable, the effects of the stress at young age do not disappear with age and shorten the lifespan of the animals.

## Additional Information

**How to cite this article**: Kogan, V. *et al.* Stability analysis of a model gene network links aging, stress resistance, and negligible senescence. *Sci. Rep.* **5**, 13589; doi: 10.1038/srep13589 (2015).

## Figures and Tables

**Table 1:** Possible lifespan extension strategies with relation to the gene network stability model parameters, and possible examples of their evolutionary deployment.

**The model parameter**	**Biological embodiment**
Coupling rate, *β*	Protective nuclear wall; transfer of mitochondrial genes to the nuclear genome
“Effective” genome size, *G*	Epigenetic inactivation of genes; tissue differentiation and specialization; temporal restriction of gene expression
Expressome (proteome, metabolome) turnover rate, *c*	Turnover/repair of proteins and metabolites: their dilution via cell division, asymmetric division; chaperones, proteasomes, and autophagosomes; metabolome turnover/maintenance; apoptosis (in multi-celled organisms)
DNA repair rate, *δ*	DNA repair; defenses against viruses and mobile genetic elements
Genotoxic stress level, *f*	Isolation from environment; suppression of ROS; dietary preferences and avoidance of noxious biomaterials; development of nocireceptors and learned responses
